# Association of MPO levels with cardiometabolic disease stratified by atherosclerotic cardiovascular risk and intensity of therapy in a workforce population

**DOI:** 10.1038/s41598-025-89373-7

**Published:** 2025-04-10

**Authors:** Olga A. Iakoubova, Farnoosh Haji-Sheikhi, Judy Z. Louie, Charles M. Rowland, Andre R. Arellano, Lance A. Bare, Charles E. Birse, Marc S. Penn

**Affiliations:** 1 Quest Diagnostics, 33608 Ortega Highway, San Juan Capistrano, CA 92675 USA; 2 Summa Health Heart and Vascular Institute, Summa Health, 525 E. Market St, Akron, OH 44304 USA

**Keywords:** Myeloperoxidase, eGFR, Liver fibrosis, Atherosclerosis, Biomarkers, Kidney

## Abstract

Cardiometabolic risk increases cardiovascular (CVD), chronic kidney (CKD) and non-alcoholic fatty liver (NAFLD) disease risk. High myeloperoxidase (MPO) levels identify individuals at risk for CVD. We whether elevation of MPO associated with kidney and liver disease risk in subgroups stratified by ASCVD risk and intensity of therapy. Adjusted logistic models assessed the associations of MPO with markers of kidney disease (estimated glomerular filtration rate) and liver fibrosis (NAFLD score > 0.676 or Fibrosis-4 [FIB-4] score > 2.67) across ASCVD risk (low < 7.5%; intermediate 7.5% to < 20%; high ≥ 20%). This retrospective study comprised 20,772 participants in an employer-sponsored health assessment. High MPO associated with impaired kidney function with low (OR 2.2, 95% CI 1.6–3.7) and intermediate (OR 2.0, 95% CI 1.3–3.5) ASCVD risk, and with high FIB-4 or NAFLD scores in low (OR 2.4, 95% CI 1.2–4.7), intermediate (OR 3.1, 95% CI 2.0–6.0), and high (OR 3.8, 95% CI 2.9–7.4) ASCVD risk groups. High MPO was associated with markers of CKD and liver fibrosis in low to intermediate ASCVD risk and treated groups. These findings demonstrate the commonality of cardiometabolic biomarkers across multiple organs.   Prospective studies are warranted to assess whether high MPO levels identify persons at risk for CKD and liver fibrosis who may benefit from preventive strategies.

**Supplementary Information:**

The online version contains supplementary material available at 10.1038/s41598-025-89373-7.

## Introduction

Cardiovascular disease (CVD), chronic kidney disease (CKD), and progressive non-alcoholic fatty liver (NAFLD) disease are highly prevalent and share common pathophysiology, with clustering of traditional cardiovascular risk factors such as dyslipidemia, hypertension, insulin resistance, and diabetes^1,2^. Chronic inflammation and oxidative stress play an important role in the pathogenesis of CVD, CKD, and NAFLD progressing to liver fibrosis, providing an additional substantial link between these diseases^1–4^. Elevated blood myeloperoxidase (MPO) levels are associated with inflammation and oxidative stress, biological processes playing a major role in the pathophysiology of chronic diseases^5,6^. MPO is a member of superfamily of heme peroxidase that is mainly expressed in neutrophils and monocytes.

A large body of evidence suggests an association of high blood levels of free MPO with increased risk for cardiometabolic and renal diseases as well as progression of NAFLD to liver fibrosis^5^. High MPO levels have been reported to identify unrecognized risk for coronary artery disease (CAD) in apparently healthy individuals^7^, risk for major coronary events in those with angiographically-defined CAD^8–11^, risk for severe CAD^12–14^, risk for primary or recurrent major CVD events in those with stable CAD^15–17^ risk for recurrent coronary events among patients after acute coronary syndrome^18^, and risk for death^19^. In previous studies, the association of MPO with cardiovascular endpoints was independent of traditional cardiovascular risk factors and independent of, or synergistic with, other risk biomarkers, including high-sensitivity C-reactive protein (hs-CRP)^18,19^ and coronary calcium score^20^. Similarly, elevated plasma MPO levels have been associated with CKD, diabetic nephropathy, and progression of NAFLD to liver fibrosis^5,21–23^. In a recent study, we demonstrated that prospectively measured MPO levels predicted risk of of 5 year mortality in a dose dependent manner due to myocardial infarction, cancer and other proinflammatory diseases^24^. We further demonstrated for the first time that lowering MPO levels resulted in a decrease in mortality^24^.

Results from multiple studies provide evidence that statin and fenofibrate therapy reduce MPO levels in patients with increased risk of atherosclerotic cardiovascular disease^25,26^ and in patients with advanced CKD^27^. Moreover, specific statins (mainly atorvastatin therapy) ameliorates NAFLD or nonalcoholic steatohepatitis (NASH), and also reduce CVD events twice as much in patients with NAFLD as in those with normal liver function^28^. In addition, therapeutic targeting of MPO has been shown to attenuate NASH in mice^29^. In a retrospective analysis of > 100,000 primary care patients with prediabetes or diabetes, knowledge of inflammatory biomarkers for vascular inflammation resulted in about a 3-fold reduction of the MPO levels during 4 years of follow-up^30^.

Given that AHA guidelines recommend increasing intensity of statin therapy and other CVD preventive therapies based on the increase in ASCVD risk^31^, greater utilization of preventive therapies would be expected in higher ASCVD risk categories. The analysis presented herein was prompted by a workplace annual health care assessment that includes testing of MPO levels and additional risk factors for CVD, CKD, and progression of NAFLD to liver fibrosis. We investigated levels of MPO according to (1) 10-year ASCVD risk, to evaluate whether high MPO identifies unrecognized risk for CKD and liver fibrosis; and (2) intensity of therapy, to evaluate whether high MPO can identify residual risk for CKD and liver fibrosis in intensively treated individuals.

## Methods

The study included all individuals who participated in an employer-sponsored annual health assessment with MPO test results who are covered by health plans provided by the employer from August 2018 to March 2021. The analysis of de-identified data was deemed exempt by the WIRB-Copernicus Group Institutional Review Board (WCG IRB) based on federal regulation 45 CFR Parts 46 and 164. The IRB similarly exempted the need for informed consent since the data had PHI removed and did not require human contact. All research was performed in accordance with institutional guidelines and regulations.

ASCVD 10-year risk for each participant was calculated using a pooled cohort equation and categorized as low (< 7.5%), medium (7.5% to < 20%), or high (≥ 20%)^32^. Participants with existing CVD based on claims data were included in the high ASCVD risk group. MPO levels were assessed using Cardio IQ^®^ Myeloperoxidase (MPO) Turbidimetric Immunoassay methodology (Quest Diagnostics Test Code: 92814). MPO levels were categorized as high (≥ 540 pmol/L), intermediate (470–539 pmol/L), or low (< 470 pmol/L)^24^. Primary analyses were designed to address relationships of high (vs. low) MPO level with markers of kidney disease (eGFR) and liver fibrosis (NAFLD fibrosis score and liver fibrosis-4 [FIB-4] score). The outcome marker of reduced kidney function was defined as eGFR < 60 mL/min/1.73m^2^. Kidney function was assessed with eGFR using the CKD-EPI creatinine Eq. 2009^33^. A composite outcome measure of high liver fibrosis score was defined as an NAFLD fibrosis score > 0.676^34^ or FIB-4 score > 2.67^35^.

The intensity of therapeutic intervention was evaluated based on refilled drug coverage claims provided by health insurance and categorized according to prior publications^36–40^. More intensive therapy was defined as the treatment of three or more risk factors for multiple cardio-renal and liver diseases or high dose single-drug therapies of drugs that have been shown to reduce both, MPO levels and risk for cardio-renal or liver disease. Less intensive therapy was defined as the treatment of 1–2 risk factors for multiple cardio-renal and liver diseases. No therapy was defined as the absence of claims for therapies that have been shown to reduce risk for cardio-renal or liver disease progression. Details of therapy categorization by drug classes are provided in the on-line supplemental material section and Supplemental Table 1.

### Statistical methods

Descriptive statistics were reported as percentages and counts for categorical data and mean and standard deviation for the continues variables. The distribution of categorical variables between groups was compared by using the Chi-square test, and the distribution of continuous variables in different groups was compared by using the Wilcoxon rank sum test. All associations of high MPO with outcome measures were assessed in comparison with low MPO; individuals with intermediate MPO levels were excluded from these analyses. Differences in the prevalence of high MPO across the ASCVD risk groups was assessed by using the chi-square trend test. Spearman’s rank correlation analysis was used to assess the correlation between MPO and BMI and hs-CRP levels.

We used logistic regression models adjusted for potential confounders including age, sex, smoking status, HDL-C and LDL-C (model 1), model 1 covariates plus hypertension, and diabetes (model 2), or model 1 covariates and hs-CRP 3 (model 3) to assess the association of high MPO levels (≥ 540 pmol/L vs. < 470 pmol/L) with reduced kidney function (eGFR < 60 mL/min/1.73m^2^) and a composite liver fibrosis score (NAFLD fibrosis score > 0.676 or FIB-4 score > 2.67). Logistic regression models were also stratified by 10-year ASCVD risk and intensity of therapy to assess the association of high MPO per outcome measure within each group. The models in this analysis were adjusted for age, sex, smoking status, HDL-C and LDL-C. For each model, the odds ratios and 95% CI are reported. Two-sided P values less than 0.05 indicated statistical significance. Analyses were conducted using the statistical software R version 4.4.0 (www.r-project.org*).*

## Results

Clinical and demographic variables of annual health assessment participants are shown in Table 1. The majority (66.4%) of the 20,772 participants were female, the mean (SD) age was 50 (11) years, and 763 (3.7%) had high MPO (Table 1). Cardiovascular risk factors such as age, sex, hypertension, diabetes, and inflammation (hs-CRP) differed significantly between high and low MPO groups (Table 1).

The distribution of participants according to kidney function (defined by eGFR) and liver fibrosis (defined by NAFLD and FIB-4 liver fibrosis score) groups across 10-year ASCVD risk is presented in Table 2. Most participants (*n* = 16,487; 79.4%) had a 10-yearASCVD risk < 7.5% (low risk); 2924 (14.1%) had a 10-year ASCVD risk of 7.5% to < 20% (intermediate risk); and 1361 (6.5%) had a 10-year ASCVD risk of ≥ 20% (high risk). Among individuals with high ASCVD risk, 14% had eGFR < 60 (vs.8% among those with intermediate ASCVD risk and 1.9% among those with low ASCVD risk), and 8.7% had NALFD > 0.676 OR FIB-4 > 2.67 (vs. 5.2% among those with intermediate ASCVD risk and 1.1% among those with low ASCVD risk) (Table 2).

The prevalence of high MPO increased significantly across ASCVD risk groups, from 3.4% in the low-risk group to 4.3% in the intermediate risk group and 4.5% in the high-risk group (*p* < 0.0003, Supplemental Fig. 1).

After adjustment for age, sex, smoking, HDL-C, and LDL-C, high MPO levels were significantly associated with impaired kidney function (OR 1.9; 95% CI 1.4–2.6) (model 1, Table 3) and with the composite outcome of liver fibrosis based on FIB-4 or NAFLD scores (OR 3.4; 95% CI 2.5–4.6). The association of high MPO with these outcomes remained significant, although moderately ameliorated, after adjustment for potential confounders such as hypertension and diabetes (model 2, Table 3) or hs-CRP (model 3, Table 3). We observed a moderate correlation between MPO and hs-CRP among participants (r^2^ = 0.3; *p* < 0.001) and a weak correlation between MPO and BMI (r^2^ = 0.045; *p* < 0.001).

**Fig. 1:**
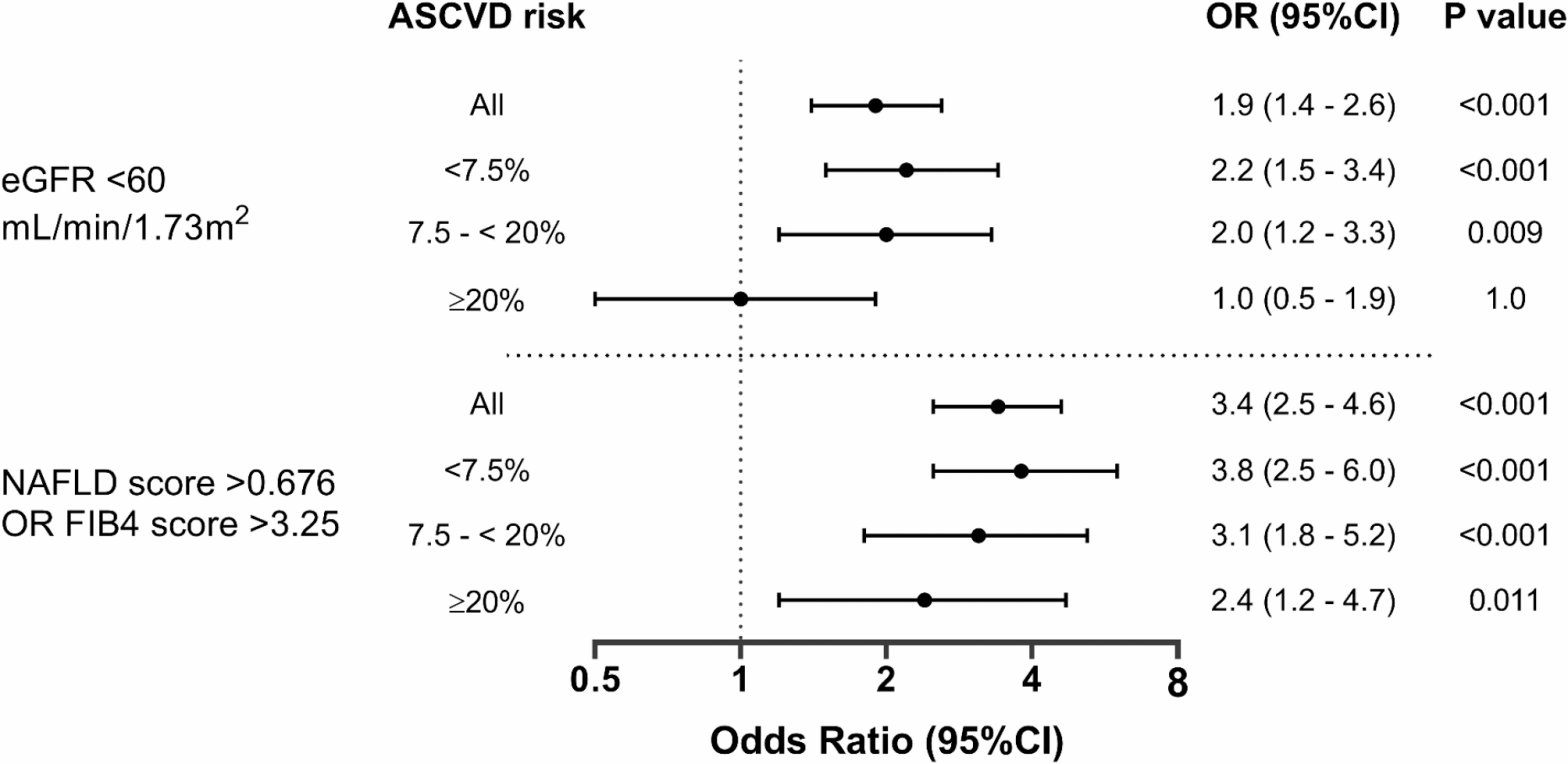
Association of high MPO with markers of impaired kidney function (eGFR < 60 mL/min/1.73 m^2^) and liver fibrosis (FIB-4 > 3.25 or NAFLD score > 0.676) according to ASCVD risk. ASCVD: atherosclerotic cardiovascular disease. High MPO: ≥540 pmol/L; low MPO: <470 pmol/L. OR adjusted for age and sex (model 1).

The associations of high MPO with outcomes stratified by ASCVD risk groups are shown in Fig. 1 and Supplemental Table 2. High MPO was significantly associated with impaired kidney function in the intermediate ASCVD risk group (OR 2.0; 95% CI 1.2–3.3) and the low ASCVD risk group (OR 2.2; 95% CI 1.5–3.4) but not in the high ASCVD risk group (OR 1.0; 95% CI 0.5–1.9) (Fig. 1 and Supplemental Table 2). However, a test of whether the association of high MPO with impaired kidney function varied significantly across ASCVD risk groups was not significant (*P* = 0.07 for interaction between MPO and ASCVD risk categories).

In all ASCVD risk groups, high MPO was substantially and significantly associated with the composite outcome of liver fibrosis (high FIB-4 or high NAFLD liver fibrosis scores), compared with low MPO, after adjusting for age, sex, smoking, HDL-C and LDL-C (Fig. 1). The association of high MPO with risk for liver fibrosis did not vary significantly across ASCVD risk groups (Fig. 1 and Supplemental Table 2). The utilization of more intensive therapy was highest (57.3%) in the high ASCVD risk group and lowest (12.2%) in the low ASCVD risk group (Table 4).

**Fig. 2:**
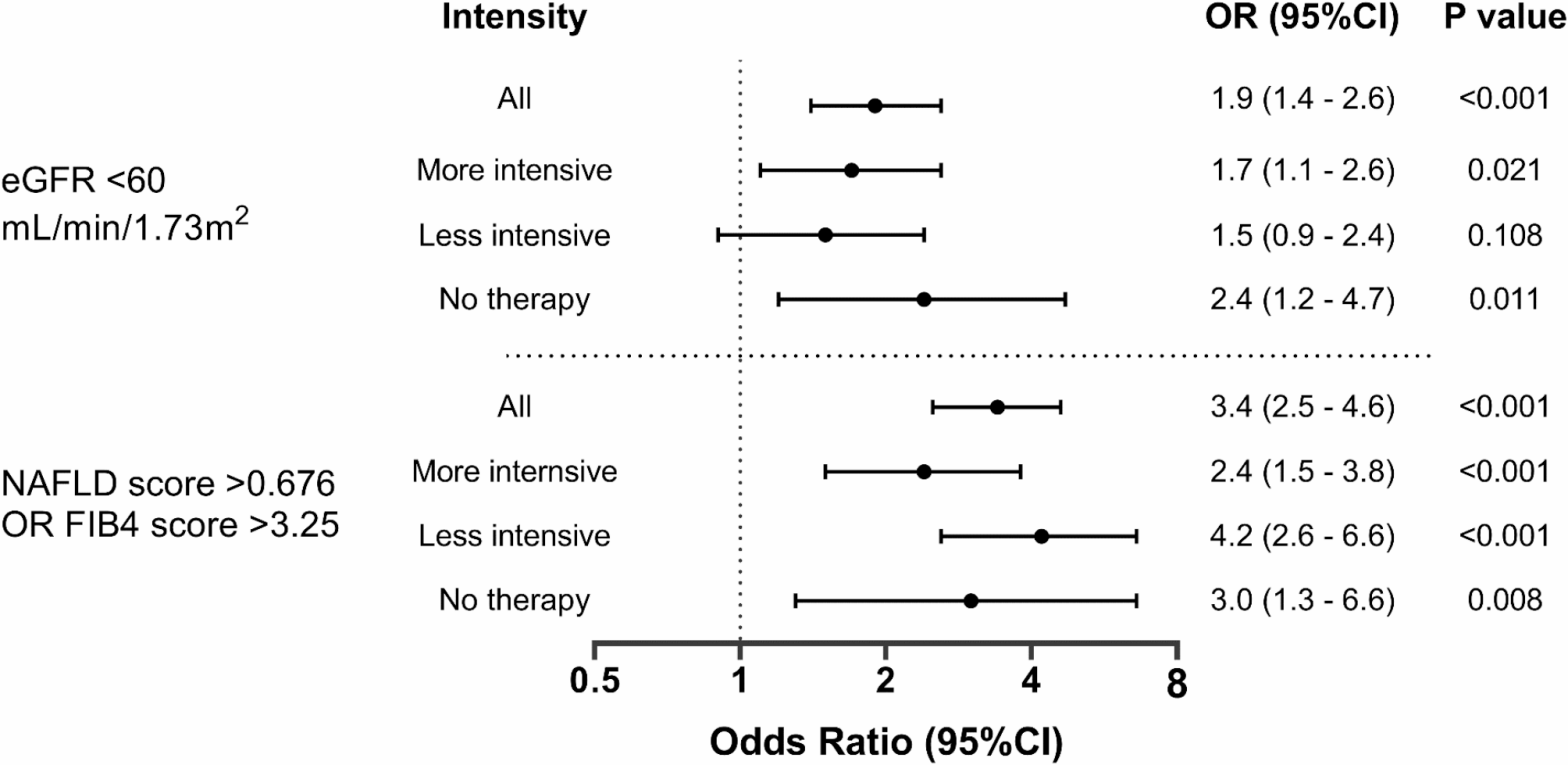
Association of high MPO with impaired kidney function and high liver fibrosis scores according to therapy intensity, compared with low MPO. High MPO ≥ 540 pmol/L; Low MPO levels < 470 pmol/L. OR adjusted for age and sex.

High MPO was significantly associated with impaired kidney function in the more intensive and the no therapy groups, but the association did not reach significance in the less intensive therapy group after adjusting for age, sex, smoking, HDL-C and LDL-C: OR was 1.7, 95% CI 1.1–2.6 in the more intensive therapy group, OR was 1.5, 95% CI 0.9–2.4 in the less intensive therapy group, and OR was 2.4, 95% CI 1.2–4.7 in the no therapy group. (Fig. 2 and Supplemental Table 3).

High MPO was also significantly associated with the composite outcome of liver fibrosis FIB-4 in all therapy-intensity subgroups, (Fig. 2). After adjusting for age, sex, smoking, HDL-C and LDL-C, OR was 2.4, 95% CI 1.5–3.8 in the more intensive therapy group, OR was 4.2, 95% CI 2.6–6.6 in the less intensive therapy group, and OR was 3.0, 95% CI 1.3–6.6 in the no therapy group. The association of high MPO with liver fibrosis did not differ between the therapy intensity groups (Fig. 2 and Supplemental Table 3).

## Discussion

In a workforce population that underwent annual health assessments with a comprehensive panel of markers, high MPO was associated with about 70% greater odds of impaired kidney function and about 2 to 3 fold greater odds of having a high liver fibrosis score, after adjusting for potential confounders. The observed association of high MPO with kidney and liver disease is consistent with previous published findings^5,21–23^. The observed odds ratios were moderately ameliorated after additional adjustment for hypertension, diabetes, or hs-CRP—risk markers shared by kidney and liver disease and ASCVD. The reduction of risk estimates after additional adjustment for potential confounders was especially pronounced for the association with liver fibrosis risk scores; these findings may be explained by the fact that diabetes is a component of the NAFLD liver fibrosis score^41^. and MPO has moderate correlation with hs-CRP. As such, adjustments for these variables may ameliorate risk estimates for the association of MPO with risk of liver fibrosis^19^. Nevertheless, the observed association of high MPO with these markers of kidney and liver diseases appear to be independent of these shared cardiometabolic risk factors.

**Table 1:** Demographic and clinical characteristics of annual health assessment participants.

Variables	High MPO (≥ 540 pmol/L)	Low MPO (< 470 pmol/L)	*P* value
	*n* = 763	*n* = 19,180	
Female, % (n)	68.7 (524)	63.6 (12,189)	< 0.001
Smoking, % (n)	11.0 (84)	10.0 (1,922)	0.4
Diabetes, % (n)	23.9 (182)	12.5(2,389)	< 0.001
Hypertension, % (n)	45.6 (348)	36.1 (6,916)	< 0.001
Age, years	50.0 ± 11.0	48.7 ± 11.1	0.002
Body mass index, kg/m	33.8 ± 8.6	29.1 ± 6.6	< 0.001
Blood pressure- diastolic, mm Hg	78.0 ± 10.7	77.3 ± 10.6	0.04
Blood pressure- systolic, mm Hg	125.1 ± 16.9	124.2 ± 16.2	0.3
LDL cholesterol, mg/dL	109.2 ± 31.7	111.6 ± 32.5	0.04
HDL cholesterol, mg/dL	52.0 ± 14.5	55.7 ± 15.0	< 0.001
HbA1c, %	6.1 ± 1.7	5.6 ± 0.9	< 0.001
Glucose, mg/dL	113.8 ± 53.4	99.5 ± 25.6	< 0.001
hs-CRP, mg/L	5.4 ± 3.6	2.9 ± 3.0	< 0.001

Data are mean ± standard deviation unless otherwise indicated. Individuals with medium MPO results (*n* = 829) are not listed.

Smoking: Current status (self-report or determined based on detection of cotinine in blood during health examination).

Diabetes: Self-reported or determined during health examination (fasting blood glucose > 125 or A1C > 6.4).

Hypertension: Self-reported or measured during health examination (systolic BP ≥ 140 or diastolic BP ≥ 90).

**Table 2:** Distribution of participants in kidney (eGFR) and liver fibrosis (FIB-4, NAFLD) groups across 10-year ASCVD risk.

			10-year ASCVD risk		
Outcome	Groups	≥ 20%	7.5 to < 20%	< 7.5%	Total
		*n* = 1,361	*n* = 2,924	*n* = 16,487	
eGFR	≥ 60	86.0% (1,171)	92.0% (2,689)	98.1% (16,167)	20,027
	< 60	14.0% (190)	8.0% (235)	1.9% (320)	745
FIB-4 score	≤ 2.67	97.8% (1,331)	98.3% (2,874)	99.6% (16,427)	20,632
	> 2.67	2.2% (30)	1.7% (50)	0.4% (60)	140
NAFLD score	<-1.455	40.3% (548)	49.3% (1,442)	81.1% (13,372)	15,362
	-1.455 to 0.676	51.8% (705)	46.5% (1,361)	18.1% (2,980)	5046
	> 0.676	7.9% (108)	4.1% (121)	0.8% (135)	364
NAFLD score > 0.676 or FIB-4 score > 2.67	No	91.3% (1,243)	94.8% (2,772)	98.9% (16,307)	20,322
	Yes	8.7% (118)	5.2% (152)	1.1% (180)	450

**Table 3:** Association of high MPO with markers of impaired kidney function (eGFR) and liver fibrosis (NAFLD, FIB-4), compared with lower MPO.

Outcome	Cases	Total	Model 1*			Model 2**			Model 3***		
			OR	95% CI	*P* value	OR	95% CI	*P* value	OR	95% CI	*P* value
eGFR <60 mL/min/1.73m^2^											
High MPO	54	763	1.9	1.4-2.6	<0.001	1.8	1.3-2.4	<0.001	1.7	1.3-2.3	<0.001
Low MPO	642	20,009	1 (ref)			1 (ref)			1 (ref)		
**NAFLD score >0.676 OR** **FIB-4 score >2.67**											
High MPO	54	763	3.4	2.5-4.6	<0.001	2.9	2.1-4.0	<0.001	2.3	1.7-3.1	<0.001
Low MPO	396	20,009	1 (ref)			1 (ref)			1 (ref)		

High MPO: ≥ 540 pmol/L; low MPO: <470 pmol/L.

*Adjusted for age, sex, smoking, HDL-C, and LDL-C.

**Adjusted for age, sex, smoking, HDL-C, LDL-C, diabetes, and hypertension.

*** Adjusted for age, sex, smoking, HDL-C, LDL-C, and hs-CRP.

Because of the numerous risk factors shared between 10-year ASCVD, CKD, and liver fibrosis, we analyzed the association of high MPO with markers of impaired kidney function and liver fibrosis in subgroups defined by 10-year ASCVD risk. High MPO was associated with about 2-fold greater odds of having markers of kidney impairment and with about 3-fold greater odds of having markers of liver fibrosis across all three 10-year ASCVD risk groups. The only exception was that high MPO was not significantly associated with impaired kidney function in the high (≥ 20%) 10-year ASCVD risk group. This finding may be explained, at least in part, by the fact that high 10-year ASCVD risk accounts for several central components of kidney disease pathophysiology, including hypertension and diabetes. The substantial association of high MPO with risk of CKD and liver fibrosis has special importance, as it could help to identify unrecognized CVD risk that is not assessed by 10-year ASCVD risk score and as such could be attributable to subclinical silent CKD and NAFLD. It has been reported that (1) CKD and NAFLD have bidirectional and synergistic relationships with CVD^42–44^, (2) the incidence and progression of CKD and NAFLD are greater in patients with CVD events^43,45^, and (3) CKD and NAFLD are independently associated with major CVD events, a leading cause of death in both CKD and NAFLD^43,46–49^.

Residual risk—the risk of CVD events remaining despite optimal management of traditional risk factors—is an important gap in both cardiovascular risk detection and preventive therapy. Indeed, the residual risk remaining after treatment is often greater than risk that is eliminated^50–53^. Residual risk is multifactorial in nature and is influenced by inflammatory pathways playing roles in many chronic diseases, including CKD and NAFLD. The results of our analysis indicated that high MPO levels were associated with markers of impaired kidney function and liver fibrosis even in the intensively treated groups: after treatments that included high-dose statins, PCSK-9 inhibitors, ACE inhibitors, and intensive glucose-lowering therapies, high MPO levels were associated with 2- to 3-fold greater odds of having markers of kidney impairment and liver fibrosis. This finding suggests that MPO can serve a valuable role in assessment of inflammatory components of the residual cardiovascular, endocrine and renal disease risk remaining after treatment of traditional risk factors. It is important to note that residual inflammatory risk can be reduced by both more aggressive conventional therapy (e.g., via pleotropic effects of potent statins, such as rosuvastatin^54^, that reduce levels of the inflammatory marker hs-CRP) and anti-inflammatory therapies such as interleukin 1-β inhibitor canakinumab, which has been reported to reduce both hs-CRP and major CVD events despite an absence of LDL-C lowering^55^. In addition, MPO has been recently proposed as a therapeutic target for cardiovascular protection^56^.

**Table 4:** Utilization of therapy according to ASCVD risk group.

	ASCVD Risk							
Therapy	≥ 20%		7.5-<20%		< 7.5%		All	
	*n* = 1,361		*n* = 2,924		*n* = 16,487		*n* = 20,772	
More intensive	57.3	(780)	37.8	(1,108)	12.2	(2,015)	18.7	(3,903)
Less intensive	27.6	(376)	33.2	(973)	22.4	(3,694)	24.3	(5,043)
No therapy	15.1	(205)	28.8	(843)	65.4	(10,778)	56.9	(11,826)

Detail of categorization for intensity of therapy is presented in Supplemental Materials and Supplemental.

Our study has a few limitations. First, the study was underpowered for testing the association of the MPO levels with markers of kidney and liver impairment in different ethnic groups and analyses are not adjusted for ethnicity, although efforts were made to offer annual health assessment to employees of different ethnicities. Second, residual risk can also be affected by suboptimal adherence to prescribed medications. As such, our analysis of the more intensively and less intensively treated groups was limited to participants who had a refill rate of at least 80% for their prescribed medications. Third, the study population includes only participants of an annual health assessment program, who may be more motivated to control their risk factors and more adherent to prescribed medications compared to general population. Finally, it is possible that a portion of the chronic hepatitis cases in this study could be due to other causes (e.g. hepatitis B, hepatitis C, alcohol intake, or autoimmune hepatitis) as we were unable to exclude such causes.

Future prospective analyses of MPO recently added to this annual health assessment population will determine whether high MPO is an independent predictor of progression of kidney disease, liver diseases, and related CVD risk, or just a marker of prevalence.

## Conclusion

The overlap of cardiometabolic risk factors for cardiac, renal, and hepatic disease suggests that we should endeavor to identify and prevent adverse kidney and liver function while we are assessing cardiovascular risk. In this workforce population, high MPO levels, compared with low MPO levels, were associated with markers of impaired kidney function and liver fibrosis. These findings identified patients at risk for cardiovascular events, CKD, and NAFLD at all stages of 10-year ASCVD risk.

## Electronic supplementary material

Below is the link to the electronic supplementary material.

Supplementary Material 1

## Data Availability

The authors will make available deidentified data used in the analyses in this manuscript upon request.
