# Supplementary material for: Association of MPO levels with cardiometabolic disease stratified by atherosclerotic cardiovascular risk and intensity of therapy in a workforce population
Source: Sci Rep. 2025 Apr 10;15:12244. doi: 10.1038/s41598-025-89373-7 (PMC11986125; doi:10.1038/s41598-025-89373-7)

Supplemental material.

Categories of therapeutic intervention intensity for association of MPO with risk of renal disease and live fibrosis according to ASCVD risk and intensity of therapy.

(1) More intensive therapy:
   a. Treatment of three or more risk factors for multiple cardio-renal and liver diseases: e.g., antihypertensives, and lipid lowering, and glucose-lowering therapies regardless of doses OR
   b. High dose single-drug therapies of drugs that have been shown to reduce both, MPO levels and risk for cardio-renal or liver disease progression (e.g., high dose statin therapy, or therapy with PCSK9 inhibitors or combination of statin with ezetimibe or fenofibrates, or the use of high-dose Pioglitazone that reduces both risk for CKD and liver fibrosis)

(2) Less intensive therapy:
   a. Treatment of 1-2 risk factors for multiple cardio-renal and liver diseases: e.g., antihypertensives, and lipid lowering, and glucose-lowering therapies regardless of doses,
   b. Multiple class or high dose single-drug therapies that have been shown to reduce a single risk for cardio-renal or liver disease progression: e.g., three or more classes of antihypertensives (ACEi, ARBs, CCB, β-blockers), or insulin or two or more glucose-lowering classes, or liver fibrosis therapy)
   c. Standard-dose statin therapy

(3) No treatment:
   a. No claims for therapies that have been shown to reduce risk for cardio-renal or liver disease progression e.g., lipid-lowering, antihypertensive (2 or less drug cases), or glucose-lowering (standard dose metformin only), or liver fibrosis therapy

Supplemental Table 1: More intensive and less intensive therapy

More Intensive therapy		Less intensive therapy	
**Drug classes**	**Drug name and daily dose**	**Drug classes**	**Drug name and daily dose**
Combination of therapies		***Combination of therapies***	
Treatment of **three or more** risk factors for multiple cardio-renal and liver diseases: e.g., antihypertensives, and lipid lowering, and glucose-lowering therapies regardless of doses	Any drug, any dose:<br>e.g., 20mg simvastatin+<br>1000 mg metformine+10mg Lisinopril	Treatment of **1-2** risk factors for multiple cardio-renal and liver diseases: e.g., antihypertensives, and lipid lowering, and glucose-lowering therapies regardless of doses	e.g., 20mg simvastatin+<br>+10mg Lisinopril<br>Or Corticosteroid therapy (effects liver fibrosis) = Valsartan/Hydrochlorothiazide.
Combination of classes		***Combination of classes***	
Ezetimibe + statin	Ezetimibe + any statin,<br>any dose	≥3 classes for BP lowering	Valsartan/Hydrochlorothiazide +Lisinopril any dose
PCSK9i+ statin	Evolocumab + any statin	≥2 classes for glucose lowering	Metformin + SU, Metformin+TZD,<br>Metformin+DPP-4 inhibitor (LINAGLIPTIN/METFORMIN HCL)
PCSK9i+ statin	Alirocumab+ any statin		
Statins+fenofibrates	Any statin + any fenofibrate, any dose		
Single class		***Single class***	
**Statins**		**Statins**	
Atorvastatin<br>Lovastatin<br>Pitavastatin<br>Pravastatin<br>Rosuvastatin<br>Simvastatin	≥20mg<br>≥20mg<br>≥4mg<br>≥40mg<br>≥10mg<br>≥20mg	Atorvastatin<br>Lovastatin<br>Pitavastatin<br>Pravastatin<br>Rosuvastatin<br>Simvastatin	<20mg<br><20mg<br><4mg<br><40mg<br><10mg<br><20mg
Fenofibrates			
Fenofibrates	≥100	Fenofibrates	<100mg
Gemfibrolzil	>600mg	Gemfibrolzil	600mg

PCSK9i			
Evolocumab	420mg/bi-weekly	Evolocumab	140mg bi-weekly or 420mg/monthly
Alirocumab	150mg/bi-weekly	Alirocumab	745mg/bi-weekly or 300mg/monthly
Thiazolidinediones*			
Pioglitazone	45mg		≤30mg
GLP-1 receptor agonists*			
Liraglutide	3.0mg/daily	Liraglutide	1.8mg/daily
Semaglutide	0.4mg/daily	Semaglutide	>0.4mg/daily
Insulin, insulin analogues	Any drug, any does		

*Single drugs addressing multiple risk factors and/or our study's endpoints: risk of liver fibrosis (progression) and glucose-lowering effect that reduce risk of CKD progression

Selected references:[36-40,57]

Supplemental Table 2: Association of high MPO with markers of impaired kidney function (eGFR) and liver fibrosis (NAFLD, FIB-4) according to ASCVD risk groups

Outcome	ASCVD risk (%)	MPO	Cases	Total	OR*	95% CI	P value
**eGFR <60 mL/min/1.73m^2**	≥20	High	10	70	1.0	0.5-1.9	1.0
		Low	180	1,291	1 (ref)		
	7.5-20	High	19	126	2.0	1.2-3.3	0.009
		Low	216	2,582	1 (ref)		
	<7.5	High	25	567	2.2	1.5-3.4	<0.001
		Low	295	15,920	1 (ref)		
**NAFLD score >0.676 OR FIB-4 score >3.25**	≥20	High	12	70	2.4	1.2-4.7	0.01
		Low	106	1,213	1 (ref)		
	7.5-20	High	18	126	3.1	1.8-5.2	<0.001
		Low	134	2,798	1 (ref)		
	<7.5	High	24	567	3.8	2.5-6.0	<0.001
		Low	156	15,920	1 (ref)		

High MPO ≥ 540 pmol/L; Low MPO levels <470 pmol/L.
*Adjusted for age, sex, smoking, HDL-C and LDL-C

Supplemental Table 3: Association of high MPO with markers of impaired kidney function (eGFR) and liver fibrosis (NAFLD, FIB-4) according to intensity of the therapy

Outcome	Intensity	MPO	Cases	Total	OR*	95% CI	P value
**eGFR <60 mL/min/1.73m^2**	More intensive	High	25	195	1.7	1.1 – 2.6	< 0.001
		Low	293	3,708	1 (ref)		
	Less intensive	High	19	258	1.5	0.9 - 2.4	0.108
		Low	255	4,785	1 (ref)		
	No therapy	High	10	310	2.4	1.2 - 4.7	0.005
		Low	143	11,516	1 (ref)		
**NAFLD >0.676 or FIB-4 score >3.25**	More intensive	High	23	195	2.4	1.5 – 3.8	<0.001
		Low	196	3,708	1 (ref)		
	Less intensive	High	24	258	4.2	2.6 - 6.6	<0.001
		Low	122	4,785	1 (ref)		
	No therapy	High	7	310	3.0	1.3- 6.6	<0.001
		Low	78	11,516	1 (ref)		

High MPO ≥540 pmol/L; Low MPO levels <470 pmol/L.
*Adjusted for age, sex, smoking, HDL-C and LDL-C

Supplemental Figure 1.

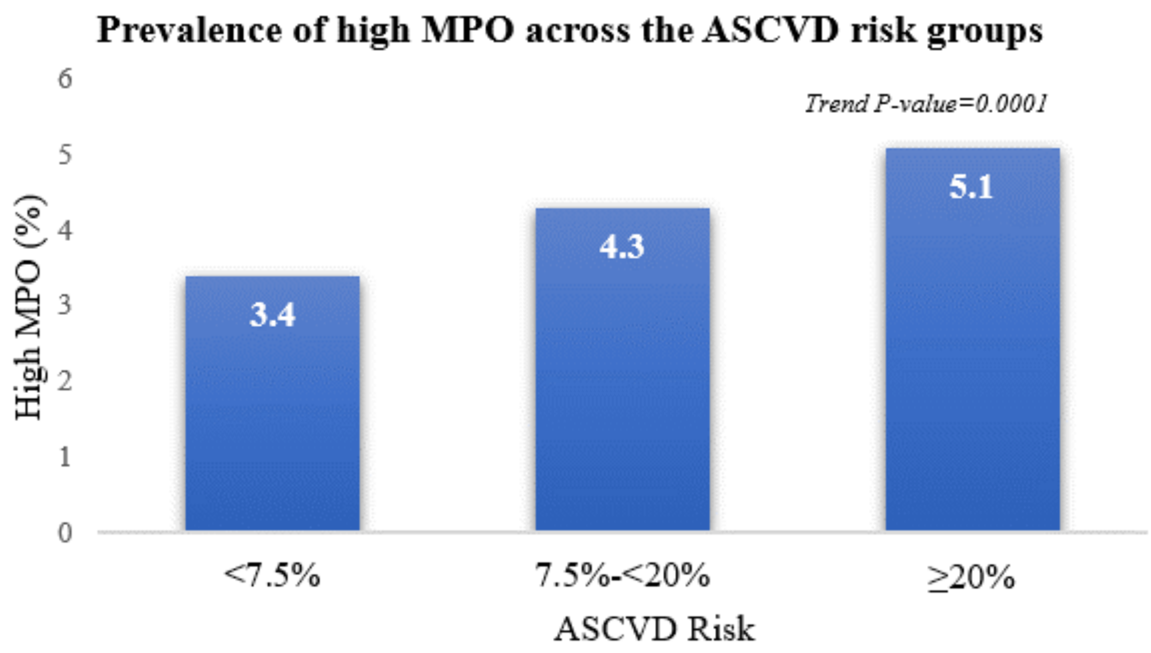

Supplement: Supplementary file 1 — Supplementary Material 1 [file 41598_2025_89373_MOESM1_ESM.pdf]
